# The histamine system and cognitive function: An in vivo H3 receptor PET imaging study in healthy volunteers and patients with schizophrenia

**DOI:** 10.1177/02698811231177287

**Published:** 2023-06-16

**Authors:** Atheeshaan Arumuham, Matthew M Nour, Mattia Veronese, Ellis Chika Onwordi, Eugenii A Rabiner, Oliver D Howes

**Affiliations:** 1 Department of Psychosis Studies, Institute of Psychiatry, Psychology & Neuroscience, Kings College London, London, UK; 2 Institute of Clinical Sciences, Faculty of Medicine, Imperial College London, London, UK; 3 Psychiatric Imaging Group, Medical Research Council, London Institute of Medical Sciences, Hammersmith Hospital, London, UK; 4 Department of Psychiatry, University of Oxford, Oxford, UK; 5 Max Planck University College London Centre for Computational Psychiatry and Ageing Research, London, UK; 6 Department of Information Engineering, University of Padua, Padua, Italy; 7 Department of Neuroimaging, Institute of Psychiatry, Psychology and Neuroscience, King’s College London, London, UK; 8 Centre for Psychiatry and Mental Health, Wolfson Institute of Population Health, Queen Mary University of London, London, UK; 9 Invicro, London, UK; 10 H Lundbeck A/s, St Albans, UK

**Keywords:** Positron emission tomography, schizophrenia, cognitive impairment associated with schizophrenia, histamine 3 receptor, executive function

## Abstract

**Background:**

The histamine-3 receptor (H3R) is an auto- and heteroreceptor that inhibits the release of histamine and other neurotransmitters. Post-mortem evidence has found altered H3R expression in patients with psychotic disorders, which may underlie cognitive impairment associated with schizophrenia (CIAS).

**Aims:**

We used positron emission tomography (PET) imaging to compare brain uptake of an H3R selective tracer between patients with schizophrenia and matched controls (healthy individuals). Regions of interest included the dorsolateral prefrontal cortex (DLPFC) and striatum. We explored correlations between tracer uptake and symptoms, including cognitive domains.

**Methods:**

A total of 12 patients and 12 matched controls were recruited to the study and were assessed with psychiatric and cognitive rating scales. They received a PET scan using the H3R-specific radioligand [^11^C]MK-8278 to determine H3R availability.

**Results:**

There was no statistically significant difference in tracer uptake between patients and controls in the DLPFC (*t*_19_ = 0.79, *p* = 0.44) or striatum (t_21_ = 1.18, *p* = 0.25). An exploratory analysis found evidence for lower volume of distribution in the left cuneus (p_FWE-corrected_ = 0.01). DLPFC tracer uptake was strongly correlated with cognition in controls (trail making test (TMT) A: *r* = 0.77, *p* = 0.006; TMT B: rho = 0.74, *p* = 0.01), but not in patients (TMT A: *r* = −0.18, *p* = 0.62; TMT B: rho = −0.06, *p* = 0.81).

**Conclusions:**

These findings indicate H3R in the DLPFC might play a role in executive function and this is disrupted in schizophrenia in the absence of major alterations in H3R availability as assessed using a selective radiotracer for H3R. This provides further evidence for the role of H3R in CIAS.

## Introduction

Schizophrenia is a debilitating condition with a prevalence of 1% and is one of the top 20 leading causes of disability worldwide (James et al., 2018). Current antipsychotics primarily act on modulating dopamine transmission and are effective in treating the positive symptoms of schizophrenia (Kaar et al., 2020). However, they have limited efficacy in the management of the negative and cognitive domains of disease, and they often have a high side-effect burden (Fusar-Poli et al., 2014; Krause et al., 2018; Leucht et al., 2009; Lobo et al., 2022). Alternative treatment options, such as increased physical activity, have been found to be therapeutic for these symptom domains but are often difficult for patients to engage with due to psychosocial barriers (Belvederi Murri et al., 2018; Stubbs et al., 2016, 2018). Cognitive impairment associated with schizophrenia (CIAS) – spanning impairment of memory, executive function and processing speed – is associated with poorer functional outcomes (Kitchen et al., 2012). This highlights the need to understand the neurobiology underlying the symptoms of schizophrenia, particularly cognitive impairments, to identify new treatment targets.

The histaminergic system, and histamine-3 receptor (H3R) in particular, has been implicated in the neurobiology of schizophrenia (Arrang, 2007). Histamine elicits its action through stimulating the G-protein coupled receptors H1R, H2R, H3R and H4R (Passani and Blandina, 2011). H3R is an autoreceptor expressed on histaminergic neuron terminals, where its activation inhibits the release of histamine (Arrang et al., 1987). Along with being a presynaptic autoreceptor, H3R is also a heteroreceptor (Ellenbroek and Ghiabi, 2014). H3R is expressed presynaptically on dopamine, glutamate, serotonin, noradrenaline, acetylcholine and GABA-ergic neurons (Clapham and Kilpatrick, 1992; Morales-Figueroa et al., 2019; Schlicker et al., 1988, 1989, 1993). Activation of H3R at these sites inhibits the release of the respective neurotransmitters. H3Rs have been localised as heteroreceptors in areas that are critical for cognitive processes including working memory and executive function, such as hippocampal circuits and corticostriatal pathway (Brown and Reymann, 1996; Doreulee et al., 2001). H3Rs are also found post-synaptically. In both rodents and humans, post-synaptic H3R density is greatest in the striatum (Martinez-Mir et al., 1990; Pollard et al., 1993), where H3Rs affect intracellular signalling of medium spiny neurons (MSN) (Ferrada et al., 2008, 2009). Post-synaptic H3Rs co-localise with D1 and D2 receptors (sometimes forming heterodimers) and act to modulate signalling in striatal circuits (inhibiting signalling of ‘direct pathway’ projections from MSN to globus pallidus, and potentiating ‘indirect pathway’ signalling from MSN to pallidum) (Ellenbroek and Ghiabi, 2014; McCutcheon et al., 2019; Panula and Nuutinen, 2013; Rapanelli et al., 2018). The functions of H3R at these different sites have highlighted the potential for H3R modulation to treat symptoms of schizophrenia, particularly cognitive impairment (Ellenbroek, 2013).

Preclinical findings support the involvement of H3R in schizophrenia. Moreover, rodents treated with scopolamine, an animal model of CIAS (Barak and Weiner, 2009), were found to have improved object recognition, and attentional set shifting when given H3R antagonists (Giovannini et al., 1999; Medhurst et al., 2007; Onodera et al., 1998; Toyota et al., 2002). These tasks are associated with both working memory and executive function (Gemkow et al., 2009). Finally, H3R antagonists have also been found to attenuate amphetamine and N-methyl-D-aspartate receptor antagonist induced hyperlocomotion and stereotypy in several studies, suggesting potential for treating positive symptoms of the disorder as well as cognitive impairments (Clapham and Kilpatrick, 1994; Faucard et al., 2006; Morisset et al., 2002).

Human studies provide further evidence for a potential role of the H3R in cognitive function and schizophrenia. A multi-modal study combining positron emission tomography (PET) with functional magnetic resonance imaging (fMRI) in healthy controls found a negative correlation between uptake of an H3R-specific tracer and the blood oxygen level dependent signal in the right dorsolateral prefrontal cortex (DLPFC), during a working memory task (Ito et al., 2018). Additionally, a post-mortem study of patients with schizophrenia found an increase in H3R expression in the DLPFC compared to healthy controls and reported a positive correlation between receptor expression and psychotic symptom severity (Jin et al., 2009).

However, to our knowledge, there has been no prior study of H3R availability in living patients with schizophrenia or other psychotic disorders, so it remains unclear whether alterations in H3R are post-mortem events or seen in vivo. In view of this, we investigated H3R expression *in vivo* in patients with schizophrenia and first episode psychosis. We hypothesised that patients would show higher expression of H3R compared to healthy controls in DLPFC (because of the post-mortem data in schizophrenia and its role in cognitive function) and striatum (given evidence implicating striatal signalling in the pathophysiology of psychotic symptoms, and the role of H3R in modulating striatal signalling) (Alfaro-Rodriguez et al., 2013; McCutcheon et al., 2019; Ryu et al., 1994, 1996). We also hypothesised that H3R expression would be positively correlated with symptom severity and negatively correlated with cognitive function.

## Methods

### Ethics statement

The study was approved by the West London & GTAC Research Ethics Committee (REC reference: 17/LO/1299) and the Administration of Radioactive Substances Advisory Committee (ARSAC licence: 630/3764/36826). Volunteers provided written informed consent to participate. We followed the Strengthening the Reporting of Observational Studies in Epidemiology (STROBE) reporting guidelines for case–control studies.

### Participants

Data were collected from 16 August 2018 until 24 March 2021. Patients were recruited from community mental health teams in London, United Kingdom. Inclusion criteria for patients were as follows: a Diagnostic and Statistical Manual of Mental Disorders (DSM-IV) diagnosis of a schizophrenia according to the *Structured Clinical Interview of DSM-IV-TR Axis I Disorders-Patient Edition* (First et al., 2002). For comparison, a sample of healthy controls matched on age (±3 years) and sex were included. An inclusion criterion for controls was no current or lifetime history of Axis I Disorder as determined by the *Structural Clinical Interview of DSM-IV-TR Axis I Disorders-Patient Edition* (First et al., 2002).

Exclusion criteria for all volunteers were as follows: history of significant head trauma (such as loss of consciousness >1 min or requiring hospital admission), dependence on illicit substances or alcohol, positive urine drug test (SureScreen Diagnostics, Derby, UK) for any illicit substances that might affect H3R (e.g. stimulants) on the day of scanning, medical comorbidity (other than minor illnesses), current or recent use (no use within 3 months) of histaminergic drugs including, but not limited to, drugs with H3 affinity such as pitolisant, and other antihistaminergic drugs), and contraindications to scanning (such as pregnancy) (see eMethods 1 in the Supplemental Material for full exclusion criteria). Antipsychotic drugs other than clozapine were permitted as they have negligible affinity for H3 receptors (Appl et al., 2012).

Participants were classified as antipsychotic-free if they had been free from antipsychotic treatment for at least 6 weeks for oral or 6 months for depot formulations, consistent with previous approaches (Jauhar et al., 2019). Antipsychotic-naïve was defined as having had no lifetime antipsychotic treatment at all.

A total of 30 participants were recruited for the study, including 16 healthy volunteers and 14 patients. Three participants (two healthy volunteers, one patient) withdrew consent prior to scanning. Of these participants who withdrew consent, the two healthy volunteers did so due to inability to find time to attend study visits, while one patient preferred not to proceed with a study involving neuroimaging. Of the remaining participants, three (two healthy volunteers, one patient) did not complete the study and withdrew following an MRI scan. From those that withdrew following the MRI scan, two were unable to attend future study visits (one healthy volunteer and one patient) while the remaining healthy volunteer was unable to tolerate the imaging process. Thus, a total of 24 participants (12 healthy volunteers, 12 patients [7 unmedicated]) completed the study by receiving both a PET and MRI scan (MR used for image co-registration and normalisation).

### Measures

#### Clinical and demographic variables

Current age and illness duration were recorded. Clinical symptom severity was determined using the Positive and Negative Syndrome Scale (PANSS) (Kay et al., 1987). Cognitive performance was assessed using the abbreviated Wechsler Adult Intelligence Scale (WAIS) to determine estimated IQ (Axelrod, 2001; Blyler et al., 2000), using the following tests: digital symbol coding, arithmetic, block design and information subtest. The dorsolateral prefrontal cortex is a key region for executive function (Nejati et al., 2018; Panikratova et al., 2020; Salehinejad et al., 2021), and impairments in executive function are a central component of the cognitive dysfunction seen in schizophrenia (Harvey, 2019). In view of this, and the post-mortem findings of altered H3R in the DLPFC in schizophrenia, we also assessed executive function using the trail making test (TMT) (for details on task, see eMethods 2 in Supplemental Material) (Periáñez et al., 2009). Detailed mapping of H3R with autoradiography has identified high density of the receptor in the hippocampus (Chazot et al., 2001; Pillot et al., 2002). These receptors regulate synaptic transmission of hippocampal circuits which may affect cognitive processes including episodic memory (Brown and Haas, 1999; Brown and Reymann, 1996; Takei et al., 2017). Therefore, we tested an exploratory hypothesis that hippocampal [^11^C]MK-8278 *V*_T_ would be inversely correlated with performance in an episodic memory task (for details of analysis and results, see eMethods 7 and eTable 3 in Supplemental Material).

Psychotropic medication history was recorded, urine drug screens were performed, and the dose of antipsychotic treatment was converted to chlorpromazine equivalent dose using previously reported methods (Leucht et al., 2014).

### Neuroimaging

#### Positron emission tomography

All participants underwent a dynamic, continuous 90-min PET acquisition after a bolus injection of [^11^C]MK-8278 (mean [SD], 263.59 [18.67] MBq), which is a radiotracer with high affinity and selectivity for H3R (Van Laere et al., 2014). All PET scans were performed between 10:00 and 13:00 to limit potential temporal confounding secondary to diurnal variation of histamine release (Brown et al., 2001). The scan was performed on a Siemens BioGraph 6 HiRez PET-CT scanner (Siemens, Erlangen, Germany). In parallel to PET imaging, continuous arterial sampling using a blood sampler (Allogg ABSS (Allogg AB, Mariefred, Sweden, http://www.allogg.se/)) was performed for the first 15 min followed by 12 discrete samples to measure radiotracer levels in blood (see eMethods 3 in the Supplemental Material for the full acquisition protocol). A low dose CT topogram (0.36 mSv) was acquired prior to PET acquisition for attenuation correction during the PET image reconstruction.

A previous study characterised [^11^C]MK-8278 uptake using 1-tissue compartmental modelling (1TCM) (Van Laere et al., 2014), but for our data set, a 2-tissue compartmental modelling (2TCM) provided superior fitting performance and lower Akaike information index estimates for the majority (77%) of the cases analysed (see eMethods 4 and eFigure 3 in the Supplemental Material). In view of this, H3R availability was determined as the [^11^C]MK-8278 volume of distribution (*V*_T_, mL/cm^3^) calculated using the standard 2TCM method with a metabolite-corrected arterial plasma input function (see eMethods 5 in the Supplemental Material for more information on PET image analysis and model validation). Prior to kinetic modelling, all the individual PET data underwent the same image processing pipeline to correct for subject motion, segment brain tissues and extract [^11^C]MK-8278 tracer activity in the main regions of interest (ROIs) using a combination of Statistical Parametric Mapping 12 (SPM12) (http://www.fil.ion.ucl.ac.uk/spm) and Functional MRI of the Brain (FMRIB) Software Library (FSL) (http://www.fsl.fmrib.ox.ac.uk/fsl) functions, as implemented in Molecular Imaging And Kinetic Analysis Toolbox (MIAKAT) (http://www.imanova.co.uk) (see eMethods 4 in the Supplemental Material for more information). The DLPFC and striatum were the primary ROIs. Secondary exploratory analyses were performed in other ROIs because of their involvement with the histaminergic system and cognition (see eTable 1 in the Supplemental Material for full list) (Alves-Rodrigues et al., 1998; Aquino-Miranda et al., 2016; Jin et al., 2009). Both the primary and exploratory ROIs described above were defined using the Clinical Imaging Centre (CIC) atlas (see eMethods 4 in the Supplemental Material for more information) (Tziortzi et al., 2011). In addition, we conducted an exploratory voxel-wise analysis to investigate whether other regions have group differences.

ROI volumes were extracted from the atlas-based segmentation of the PET and MRI images. Briefly, the MRI template was nonlinearly registered to the subject’s brain MRI and the resulting deformation field saved. The latter was then applied to the CIC atlas to provide a brain individual anatomical segmentation for both PET and MRI images. These steps were implemented in MIAKAT as part of standard image pre-processing. Exploratory, whole-brain, voxel-wise analysis was conducted using SPM12, to determine whether there were alterations in *V*_T_ in brain regions outside of ROIs, using [^11^C]MK-8278 *V*_T_ parametric maps derived using the Logan graphical approach (Logan et al., 1990).

The main outputs from the image analysis were manually quality controlled (QC) and image analysis was blind to diagnosis status (for full description see eMethods 4 PET image analysis in the Supplemental Material). Scans were excluded blind to diagnosis on the basis that there were still obvious artefacts that could not be corrected in postprocessing (e.g. presence of within frame motion artefacts). All scans passed QC assessment.

#### Magnetic resonance imaging

All subjects underwent structural MRI to facilitate the anatomical delineation of ROIs. T1-weighted three-dimension magnetisation-prepared rapid acquisition gradient echo (MPRAGE) images were acquired on a Siemens Magnetom Verio Syngo MR B17 3T scanner (Siemens, Erlangen, Germany) according to the following parameters: repetition time = 2300.0 ms, echo time = 2.98 ms, flip angle = 9°, field of view (FOV) = 256 × 256 mm, 160 sagittal slices of 1-mm thickness, distance factor = 50%, voxel size = 1.0 × 1.0 × 1.0 mm.

### Statistical analysis

Power calculations to determine sample sizes were performed using the G*Power 3.1 software (ver. 3.1.9.7; Heinrich-Heine-Universität Düsseldorf, Düsseldorf, Germany) (Faul et al., 2007, 2009). As no previous study has used the radioligand [^11^C]MK-8278 to assess potential group differences between patients with schizophrenia and healthy volunteers, we instead used a previous post-mortem study to estimate effect sizes (Jin et al., 2009). This study identified a significant difference between groups with a large effect size of Cohen’s *d* = 1.37 (patients: mean ± SD = 2.37 ± 0.49, controls: mean ± SD = 1.74 ± 0.43). This effect size would require a total sample size of 20 participants, with 10 in each group, to detect a significant difference between groups (α = 0.05, two-tailed).

Statistical Product and Service Solutions (SPSS) version 22 (IBM Corp) was used for all statistical analyses and the significance level was set to *p* < 0.05 (two-tailed). Data normality was assessed using the Shapiro-Wilk test. Categorical clinical, demographics and experimental variables were compared across groups using χ^2^ tests; and continuous variables were assessed using independent samples t-tests and Mann-Whitney tests for parametric and non-parametric data, respectively. We assessed for the presence of outliers using the Tukey method within SPSS (Tukey, 1977).

To determine whether tracer uptake was higher in patients as compared to controls, a repeated measures analysis of variance (ANOVA) was performed to examine the main effect of diagnosis and diagnosis × ROI interaction. Following this, independent samples t-test or Mann-Whitney tests were used to examine mean regional differences for normally and non-normally distributed data, respectively.

To assess whether regional volume affected tracer uptake, we used Pearson’s or Spearman’s correlation, for normally and non-normally distributed data, respectively. A separate analysis of covariance (ANCOVA) was then conducted with each specific ROI as the dependent variable and group (patient or control) as the independent variable, with the specific ROI volume included as a covariate. This analysis determines whether regional volume had a significant effect on group differences for specific ROIs.

Group level between subjects variability was measured using the coefficient of variation for the *V*_T_ in the ROIs by calculating the ratio of the standard deviation to the mean.

An exploratory, whole-brain, voxel-wise analysis was conducted using SPM12. Here, an independent sample t-test was used to investigate whether there were group differences in the *V*_T_ of [^11^C]MK-8278 at each brain voxel, family-wise corrected for multiple comparisons using random field theory as implemented in SPM12 (peak-level family-wise error corrected *p* value < 0.05 deemed statistically significant).

To investigate our hypothesis that DLPFC and striatum *V*_T_ is positively correlated with total symptom severity, and negatively associated with estimated IQ, Pearson’s or Spearman’s correlation coefficients were calculated for normally and non-normally distributed data, respectively. Pearson’s and Spearman’s correlation were also employed for testing the relationship between DLPFC *V*_T_ and time needed to complete TMT tasks (longer duration indicating worse performance). For comparison of correlation coefficients between patients and controls, we performed a z-test on Fisher r-to-z transformed correlation coefficients, which allows the comparison of the correlation coefficients of an independent variable with a set of dependent variables (Hinkle et al., 1988). As H3R localised in the striatum may affect the direct and indirect pathways, we also determined whether striatal tracer uptake was correlated with TMT performance, to elucidate whether striatal H3R’s effect on motor function could impact TMT performance (Ellenbroek and Ghiabi, 2014). We also performed secondary analyses to explore whether there were relationships with the general, positive and negative subscales. Bonferroni corrections were used for all analyses to yield appropriate family-wise error-corrected significance thresholds.

## Results

### Demographics and experimental variables

We found no significant differences between groups for age or weight, and sex was matched exactly (see Table 1). There was a significant difference between groups in estimated IQ (control mean [SD] = 110.9 ± 16.8, patient mean = 91.6 ± 12.5, t_22_ = 3.20, *p* = 0.004). There was also a significant difference between groups for the injected radiotracer dose, with patients on average receiving 9% less than controls. However, there was no significant correlation between injected dose and *V*_T_ values (see eTable 2 in the Supplemental Material for details on scanning parameters).

**Table 1.** Demographics and experimental variables.

Characteristics	Healthy volunteers (*n* = 12)	Patients (*n* = 12)	*t*	*p*
Sex, male:female	9:3	9:3	*X*^2^ = 0	1.00
Age, years, mean (SD)	30.3 (11.5)	30.4 (13.0)	Mann-Whitney *U* = 67.50	0.80
Weight, kg, mean (SD)	73.1 (12.4)	79.7 (13.4)	−1.26	0.22
Injected dose, MBq (SD)	275.6 (10.7)	251.5 (18.1)	Mann-Whitney *U* = 11.00	<0.01
Antipsychotic-free (*n*)		7		
Chlorpromazine equivalent dose/mg per day, mean (SD)		324.3 (118.2)		
PANSS positive, mean (SD)		16.8 (3.7)		
PANSS negative, mean (SD)		17.7 (3.9)		
PANSS general, mean (SD)		35.4 (7.5)		
PANSS total, mean (SD)		69.8 (12.7)		
Estimated IQ, mean (SD)	110.9 (16.8)	91.6 (12.5)	3.20	<0.01
TMT Part A/s, mean (SD)	21.2 (6.1)	34.4 (7.7)	−4.67	<0.01
TMT Part B/s, mean (SD)	47.8 (17.9)	95.3 (28.9)	Mann-Whitney *U* = 132.50	<0.01

IQ: intelligence quotient; n: number; PANSS: Positive and Negative Syndrome Scale; SD: standard deviation; TMT: trail making test.

Scores range for total PANSS = 53–88, general PANSS subscale: 25–46, positive subscale of the PANSS: 12–27; negative subscale of the PANSS: 8–28; and depression item: 1–6. Higher scores indicate greater symptom severity.

Scores range for total PANSS = 53–88, general PANSS subscale: 25–46, positive subscale of the PANSS: 12–27; negative subscale of the PANSS: 8–28; and depression item: 1–6. Higher scores indicate greater symptom severity.

### Tracer uptake in a priori ROIs

**Figure 1.**
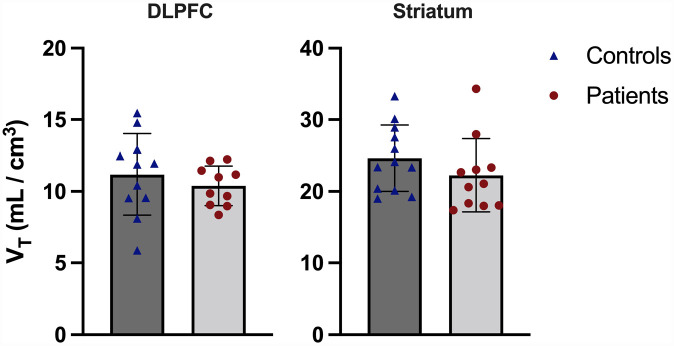
Tracer uptake in dorsolateral prefrontal cortex (DLPFC) and striatum for controls and patients. There was no significant difference between groups in either region. Error bars show ±1 standard deviation. *V*_T_, volume of distribution.

Figure 1 shows the volume of distribution data by group for both primary ROIs. DLPFC and striatum *V*_T_ data were normally distributed (*p* = 0.93 and *p* = 0.07, respectively), and no outliers were present. There was no statistically significant effect of group on *V*_T_ (DLPFC: control mean = 11.2 ± 2.8, patient mean = 10.3 ± 1.4, striatum: control mean = 24.6 ± 4.6, patient mean = 22.3 ± 5.1, main effect of group F_1,19_ = 1.07, *p* = 0.31, repeated measures ANOVA) and no significant group-by-ROI interaction effect (F_1,19_ = 0.52, *p* = 0.48). The coefficients of variation for patients and controls were 13.3% and 25.4%, respectively, in the DLPFC, while they were 22.9% and 18.7%, respectively, in the striatum. DLPFC and striatal volume were normally distributed (*p* = 0.44 and *p* = 0.11, respectively), and no outliers were present. There was no significant correlation between DLPFC volume and *V*_T_ (*r* = –0.14, *p* = 0.55, Pearson’s correlation, two-tailed), nor striatal volume and *V*_T_ (*r* = –0.13, *p* = 0.56, Pearson’s correlation) in the combined sample. Neither DLPFC (F_2,20_ = 0.52, *p* = 0.60) nor striatal (F_2,22_ = 1.39, *p* = 0.27) volume had a significant effect of group on ROI tracer uptake when included as a covariate in ANCOVA.

### Group comparisons for voxel-wise data and exploratory ROIs

**Figure 2.**
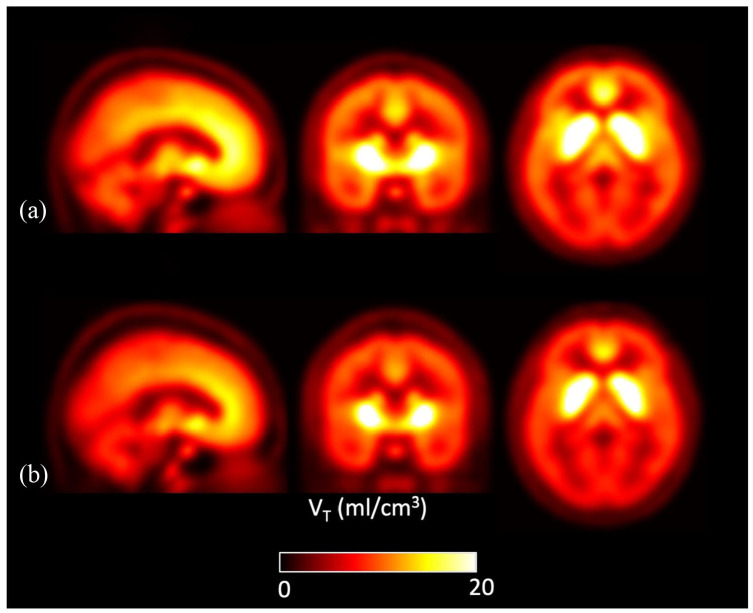
Mean parametric [^11^C]MK-8278 *V*_T_ brain images for the patient (*n* = 12) and control (*n* = 12) groups: (a) healthy controls and (b) patients.

**Figure 3.**
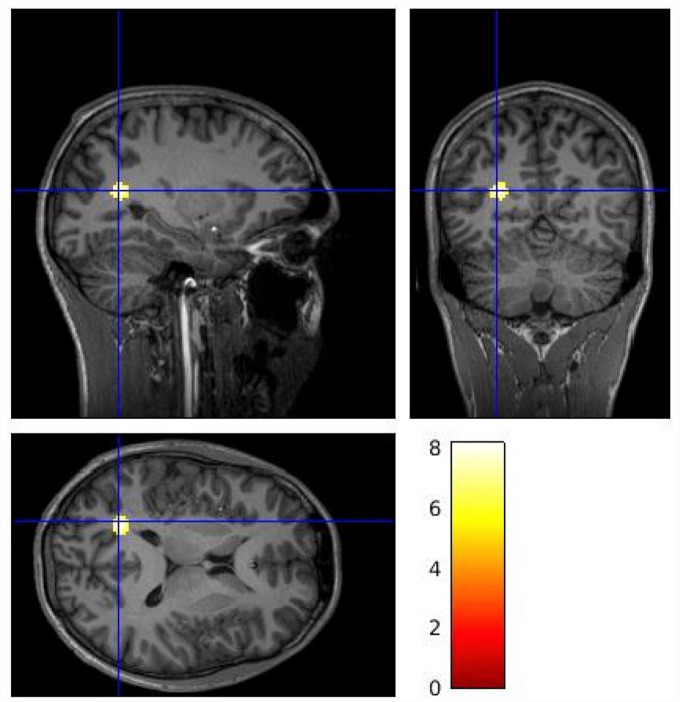
Voxel-wise analysis [^11^C]MK-8278 *V*_T_ brain images identifying lower tracer binding in patients compared to controls (control > patient contrast). The gradient heat bar indicates T contrast from a voxel-wise two sample *t*-test. The cluster included voxels within the left cuneus (peak MNI coordinates *x* = −20, *y* = −62, *z* = 16, p_FWE corr_ = 0.01).

Figure 2 shows parametric images for mean *V*_T_ values at the voxel level by group across the whole brain. The voxel-wise analysis identified lower [^11^C]MK-8278 binding in patients compared to controls in a voxel cluster with its peak in the left cuneus (Figure 3; p_FWE corr_ = 0.01, peak-level significance, controls > patients contrast). When applying the patients > controls contrast, no significant difference was revealed at cluster or voxel level.

There were no significant group differences in the hippocampus, thalamus, frontal lobe, parietal lobe, occipital lobe, anterior cingulate cortex, insular cortex or pons ROIs (see eTable 1 in the Supplemental Material for *V*_T_ data of exploratory regions). Although there was a group difference in *V*_T_ in the nucleus accumbens, this did not survive correction for multiple comparisons (Bonferroni adjusted α = 0.05/9 = 0.006; control mean = 28.1 ± 3.8, patient mean = 23.8 ± 3.4, t_18_ = 2.68, *p* = 0.015).

### Tracer uptake and symptoms

PANSS total symptom severity was normally distributed (*p* = 0.43). We found no significant association between PANSS total symptom severity and DLPFC *V*_T_ (*r* = 0.17, *p* = 0.64, Pearson’s correlation), or striatal *V*_T_ (*r* = 0.15, *p* = 0.67, Pearson’s correlation). Secondary analyses to determine association between ROIs tracer uptake and positive, negative and general PANSS subscales found no significant relationship (see eFigures 4–6 in the Supplemental Material).

Estimated IQ was normally distributed (*p* = 0.37). We found no association between IQ and tracer uptake in the DLPFC (patients: *r* = 0.23, *p* = 0.53, controls: *r* = –0.34, *p* = 0.30, Pearson’s correlations) or striatum (patients: *r* = 0.14, *p* = 0.68, controls: *r* = 0.04, *p* = 0.90, Pearson’s correlations).

**Figure 4.**
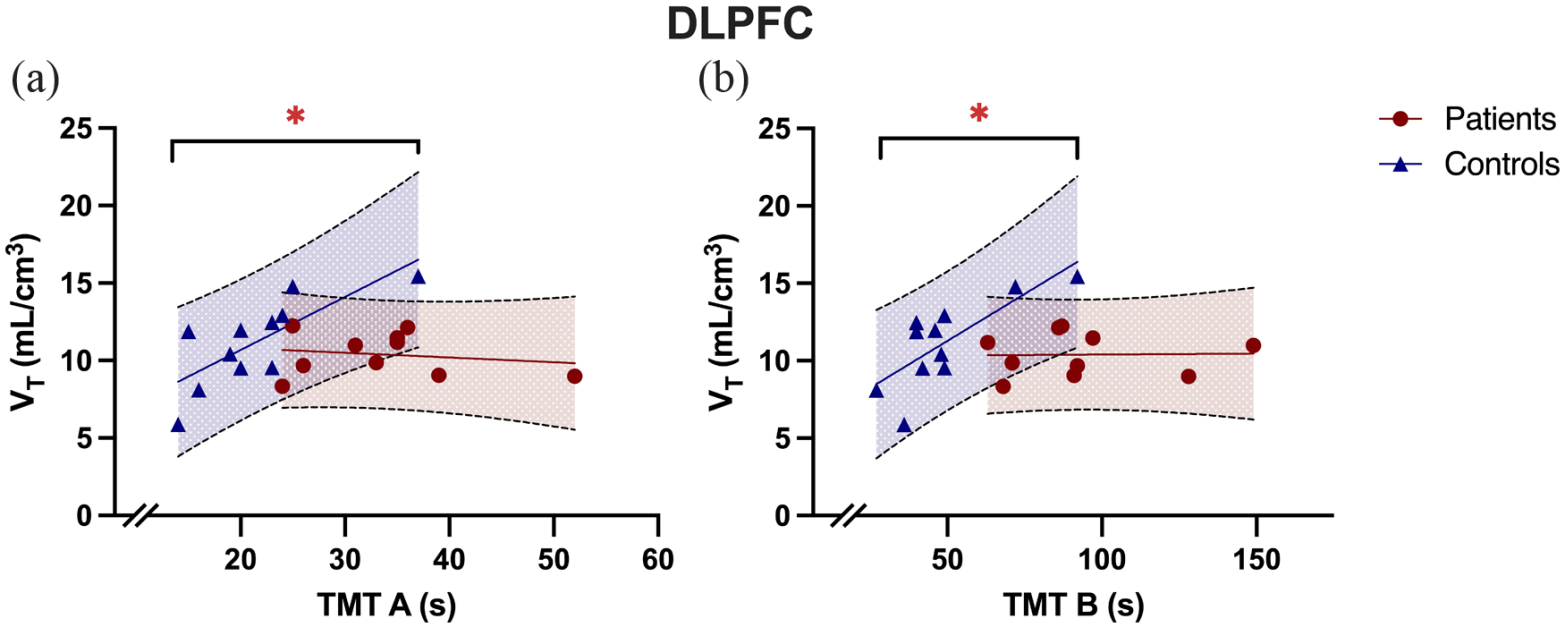
Tracer uptake in dorsolateral prefrontal cortex (DLPFC) correlated with TMT performance in controls but not patients. (a) The image shows DLPFC *V*_T_ correlated with TMT A performance in controls (*r* = 0.77, *p* = 0.01) but not in patients (*r* = −0.18, *p* = 0.62; *r* dif = 0.95, *z* = 2.30, 95% CI = 0.18–0.98, *p* = 0.02 ), while (b) shows DLPFC *V*_T_ correlated with TMT B performance in controls (rho = 0.74, *p* = 0.01) but not in patients (rho = −0.06, *p* = 0.81; *r* dif = 0.79, *z* = 1.92, 95% CI = −0.02–0.97, *p* = 0.06). s, seconds; *V*_T_, volume of distribution. *p < 0.05.

While TMT A was normally distributed (*p* = 0.17), TMT B was not (*p* < 0.05). We found a significant group difference in time taken to complete both TMT A (control mean = 12.2 ± 6.1, patient mean = 34.4 ± 7.7, t_22_ = –4.67, *p* < 0.001, independent sample t-test, two-tailed) and TMT B (control mean = 47.8 ± 17.9, patient mean 95.3 ± 28.9, *U* = 132.50, *z* = 3.50, *p* < 0.001, Mann-Whitney U test, two-tailed). In patients, we found no association between TMT A performance (where a higher value for TMT A indicates worse performance) and tracer uptake in the DLPFC (Bonferroni adjusted α = 0.05/4 = 0.0125; *r* = –0.18, α = 0.62, Pearson’s correlation). Similarly, we found no association between TMT B performance in patients and tracer uptake in DLPFC (rho = –0.06, α = 0.81, Spearman’s correlation). In controls, we found a significant correlation between TMT A and tracer uptake in the DLPFC (Figure 4; *r* = 0.77, α = 0.006, Pearson’s correlation) a similar significant correlation between TMT B performance in controls and tracer uptake in the DLPFC (rho = 0.74, α = 0.010, Spearman’s correlation).

We found a significant difference when comparing the correlation coefficients between patients and controls for the relationship between DLPFC *V*_T_ and TMT A performance (*r* dif = 0.95, *z* = 2.30, 95% CI = 0.18–0.98, *p* = 0.02), and a trend towards a significant difference for TMT B performance (*r* dif = 0.79, *z* = 1.92, 95% CI = –0.02–0.97, *p* = 0.06).

We found no significant relationship between striatal *V*_T_ and TMT A (patients: *r* = –0.17, *p* = 0.61, controls: *r* = 0.13, *p* = 0.68) or TMT B performance (patients: rho = –0.14, *p* = 0.69, controls: rho = 0.01, *p* = 0.97).

## Discussion

We found no significant difference between groups in [^11^C]MK-8278 *V*_T_ in the DLPFC or striatum ROIs, but it was lower in patients with schizophrenia than controls in the left cuneus in the exploratory whole-brain analysis. Although we did not find a correlation between ROI *V*_T_ and psychosis symptom severity (measured by PANSS), our results indicate a positive correlation between TMT performance and DLPFC *V*_T_ in healthy volunteers which is absent in the patient group. Further exploratory analysis found a non-significant relationship between hippocampal *V*_T_ and episodic memory, assessed via the Rey Auditory Verbal Learning Test (for results, see eTable 3 in Supplemental Material) (Schmidt, 1996).

These findings run counter to findings from a post-mortem study that identified significantly higher level of H3R expression in the DLPFC (Jin et al., 2009). In this post-mortem study, the majority of the 15 subjects analysed were medicated (*n* = 12), and the authors found that antipsychotic treatment was associated with higher radioligand binding in absolute terms, although they did not find a significant difference between medicated and unmedicated subjects. Furthermore, five of the medicated participants included in the post-mortem study were treated with clozapine which is known to have a higher affinity for H3R (Ki = 1000 nM) compared to other antipsychotics (National Institute of Mental Health’s Psychoactive Drug Screening Program (PDSP) database (https://pdsp.unc.edu/databases/kidb.php) (Kathmann et al., 1994). In our patient group, the majority of subjects were unmedicated (*n* = 7, 60%), and those who were medicated were treated with either olanzapine or risperidone, which have negligible affinities for H3R (olanzapine and risperidone Ki for H3R > 100,000 nM) (Appl et al., 2012). Thus, antipsychotic exposure in the post-mortem study may explain its finding of higher H3R in the DLPFC in schizophrenia, and the discrepancy with our finding of no significant difference in this region relative to controls. Our findings, also extend the post-mortem work by examining other brain regions, finding no evidence of alterations, other than lower levels in schizophrenia in a region including the left cuneus. Although there is a paucity of information regarding the role of H3R in the cuneus, evidence indicates abnormal cuneus function in schizophrenia is associated with early impairments of cognitive function, including attention and working memory (Seymour et al., 2013; Yan et al., 2020). Thus, the finding of lower H3R in this region could indicate a neuroreceptor mechanism contributing to abnormal cuneus function in schizophrenia. However, given this finding arose from an exploratory voxel-wise analysis, it warrants replications before firm conclusions can be drawn.

Our finding that higher DLPFC *V*_T_ was strongly associated with poorer TMT performance in controls provides, to our knowledge, the first in vivo evidence that H3R density is associated with cognitive task performance in humans. This extends prior evidence that H3R antagonists improve cognitive measures in rodents (Sadek et al., 2016; Vohora and Bhowmik, 2012), to show a relationship with uptake of an H3R ligand and cognition in humans. High constitutive activity of H3R inhibits the release of histamine as well as other procognitive neurotransmitters such as dopamine, glutamate and acetylcholine in cortical regions (Arrang et al., 2007; Baronio et al., 2014), which may account for the relationship we observe. Our finding also suggests that H3R antagonists could be effective in improving cognitive function. Although no studies are available examining the effect of H3R antagonists on TMT performance in humans, studies in rodents have found H3R antagonists improves performance in tasks employing working memory and executive function, which are both cognitive processes involved in the TMT (Giovannini et al., 1999; Medhurst et al., 2007; Onodera et al., 1998). The TMT assesses executive function, but also has a motor component, with previous studies suggesting that poorer performance by patients with schizophrenia could be due to psychomotor retardation associated with the condition (Wölwer and Gaebel, 2002). H3R alterations could theoretically affect motor function by inhibiting signalling of the D1R-expressing direct pathway while potentiating signalling of the D2R-expressing indirect pathway (Ellenbroek and Ghiabi, 2014; McCutcheon et al., 2019; Panula and Nuutinen, 2013; Rapanelli et al., 2018) and so underlie our findings of group differences in TMT performance. However, our additional analyses found no significant relationship between striatal *V*_T_ and TMT performance, suggesting that striatal H3R availability does not account for the group differences in TMT performance.

### Strengths and limitations

To our knowledge, this is the first study exploring H3R expression in vivo in patients with psychotic disorders. [^11^C]MK-8278 has a nanomolar affinity for H3R and Test-Retest (T-RT) repeatability of ~5% in most regions (Van Laere et al., 2014). When evaluated in over 170 receptor binding or enzyme assays, the compound was found to be selective for H3R (Ki = 0.54 nM), with only weak off-target binding found at 5-HT2 (51% inhibition at 10 μM) and 5-HT2A (62% inhibition at 10 μM) (Van Laere et al., 2014). However, Van Laere et al. found increased variation of uptake in the caudate, where repeatability was 20%. This may be a limitation when interpreting tracer uptake in the striatum. In a previous paper that evaluated [^11^C]MK-8278, the authors used a simple 1TCM (*V*_T_ = k1/k2) rather than the 2TCM. In the analysis of our data, we found that 2TCM provided a superior description of tracer uptake. Estimated time-activity curves (TAC) were developed using these models, with the 2TCM having a much closer fit to the observed PET TAC, thereby providing a more accurate model with a minimised sum-of-squares (Gunn et al., 2002) (see eFigure 3 in the Supplemental Material for TAC modelling). Other strengths include that the PET acquisition was performed during a similar period of time for all subjects, thereby limiting potential temporal confounding secondary to diurnal variation of histamine release (Brown et al., 2001).

A potential limitation is that the study was not powered to detect small group differences. The possibility of a Type I or II error should be considered when evaluating our findings of a relationship between TMT performance and DLPFC *V*_T_ in healthy volunteers, and no significant group differences in *V*_T_ in primary ROIs. Furthermore, due to the exploratory nature of our findings in the voxel-wise analysis and correlations with cognitive performance, further studies with larger sample sizes would be beneficial to gain a greater understanding of the nature of these relationships. (Section including the power calculation as per reviewers 1 and 3). It should also be noted that patients received a 9% lower dose of [^11^C]MK-8278 compared to controls, which may have an effect on tracer uptake (control mean = 275.63 MBq ± 10.29, patient mean = 251.55 MBq ± 17.36, *U* = 11.00, *p* < 0.001). However, further analysis indicated that there was no significant correlation between injected dose and primary ROIs *V*_T_, indicating that this variation in injected dose did not have a significant effect on tracer uptake (DLPFC, Spearman’s rho = 0.16, *p* = 0.49; striatum, rho = 0.09, *p* = 0.67).

The possibility that antipsychotics used by some of the patients may bind to H3R should also be considered. However, the antipsychotics used by our patient group have a negligible affinity for H3R, and as the tracer is specific to the receptor, it is unlikely that antipsychotics have a direct effect on tracer binding (Van Laere et al., 2014). Nevertheless, both antipsychotics that patients were taking in this study (i.e. olanzapine and risperidone) are known to have higher affinities for H1R (Appl et al., 2012). From the observed data, it can be hypothesised that H1R antagonism by these antipsychotics might cause downstream alterations of H3R expression, through compensatory mechanisms due to increase in histamine synthesis and release. However, a study that examined the effect of sub-chronic olanzapine exposure in rats, found that although H1R expression and histamine release was increased, there was not a significant effect on H3R expression (He et al., 2017). Thus, antipsychotic exposure is unlikely to confound our findings, although we cannot completely rule out an effect.

### Implications of our findings

There was no association between tracer uptake in primary ROIs of patients and PANSS symptom severity measures. This indicates that H3R levels are unlikely to underlie schizophrenia symptoms and may explain why it has proven challenging to develop medications targeting H3R to treat schizophrenia (Egan et al., 2013; Haig et al., 2014; Jarskog et al., 2015).

Our findings that the relationship between H3R expression and TMT performance found in controls is not present in patients with schizophrenia suggests that disruption of the histaminergic system in schizophrenia may contribute to some cognitive symptoms in schizophrenia, although it should be recognised that this lack of association does not prove causality. In contrast, we did not find associations between IQ and H3R *V*_T_ in the ROIs. The abbreviated WAIS employed in this study to measure IQ and delivered four cognitive assessments including digital symbol coding, arithmetic, block design and information subtest. Although these assessments include testing processing speed, they do not explicitly test executive function, which may underlie the lack of association seen between [^11^C]MK-8278 tracer binding and IQ (Buczylowska and Petermann, 2017; Wechsler, 1955). Thus, our findings could suggest that H3Rs are more important in regulating executive than other cognitive processes.

Despite promising effects of H3R antagonists to correct cognitive deficits in preclinical models, which included executive function paradigms, these compounds have borne mixed results in treating CIAS in clinical trials (Egan et al., 2013; Haig et al., 2014; Jarskog et al., 2015; Vohora and Bhowmik, 2012). Given the disruption of H3R expression in patients with schizophrenia, our findings suggest that future studies should focus on examining the potential ameliorating effect of H3R antagonists on executive function. Nevertheless, a double-blind randomised controlled trial found high dose betahistine, a H1R agonist/H3R antagonist, significantly improved cognitive function in patients with schizophrenia, including verbal and visual learning domains (Wang et al., 2021), while a betahistine challenge examined through fMRI identified modulation of task-evoked activity (van Ruitenbeek and Mehta, 2013). Thus, taken together these findings indicate disruption of the wider histaminergic system, including both H1R and H3R, could contribute to cognitive dysfunction in schizophrenia, although further work is needed to confirm this.

## Conclusions

Our findings provide evidence for an association between DLPFC H3Rs and executive function in controls, and that this association is disrupted in schizophrenia. We found no evidence for major alterations in H3R levels in this or most other brain regions. Our findings suggest potentially altered histaminergic regulation of executive function in schizophrenia, in the absence of major changes in receptor levels. Given the limitations of this work, future studies are required to detail the relationship between H3R and specific cognitive domains. Nevertheless, these results build upon a body of evidence demonstrating the potential amelioration of cognitive dysfunction in neuropsychiatric conditions through modulation of the histaminergic system.

## Supplemental Material

sj-docx-1-jop-10.1177_02698811231177287 – Supplemental material for The histamine system and cognitive function: An in vivo H3 receptor PET imaging study in healthy volunteers and patients with schizophrenia Click here for additional data file. Supplemental material, sj-docx-1-jop-10.1177_02698811231177287 for The histamine system and cognitive function: An in vivo H3 receptor PET imaging study in healthy volunteers and patients with schizophrenia by Atheeshaan Arumuham, Matthew M Nour, Mattia Veronese, Ellis Chika Onwordi, Eugenii A Rabiner and Oliver D Howes in Journal of Psychopharmacology
