# Supplementary material for: The histamine system and cognitive function: An in vivo H3 receptor PET imaging study in healthy volunteers and patients with schizophrenia
Source: J Psychopharmacol. 2023 Jun 16;37(10):1011–22. doi: 10.1177/02698811231177287 (PMC10612380; doi:10.1177/02698811231177287)
Supplement: sj-docx-1-jop-10.1177_02698811231177287 – Supplemental material for The histamine system and cognitive function: An in vivo H3 receptor PET imaging study in healthy volunteers and patients with schizophrenia [file sj-docx-1-jop-10.1177_02698811231177287.docx]

**Histamine 3 and psychotic disorders: supplementary information**

**eMethods 1:** Full Inclusion and Exclusion Criteria

**eMethods 2:** TMT administration

**eMethods 3:** PET Imaging Acquisition Parameters

**eMethods 4:** PET Image Analysis: Preprocessing Methods

**eMethods 5:** [^11^C]MK-8278 Uptake: Kinetic Modelling Validation

**eMethods 6:** Movement Parameters

**eMethods 7:** [^11^C]MK-8278 blood uptake: cross-sectional comparison

**eMethods 8:** Exploratory analyses

**eTable 1:** PET data

**eTable 2**. Experimental Parameters

**eTable 3:** RAVLT performance

**eTable 4:** Episodic memory and hippocampal [^11^C]MK-8278 V_T_

**eFigure 1:** Whole blood and parent plasma data of radiotracer activity over time

**eFigure 2:** POB activity concentration ratio and PPf activity over time

**eFigure 3:** TAC for representative control and patient, including 1TCM and 2TCM

**eFigures 4-6:** ROI [^11^C]MK-8278 V_T_ and symptoms

**eReferences**

**eMethods 1**. Full Inclusion and Exclusion Criteria

Inclusion criteria for all participants: 1) between 18­65 years of age; 2) capacity to consent to participation in the study; 3) modified Allen’s test showing adequate collateral circulation to the hand (to ensure safety of radial arterial catheterization); 4) no history of clotting or renal abnormality and no abnormal blood results on screening blood test; 5) English speaking sufficient to understand task instructions and information sheet; 6) in females, a negative urine pregnancy test at screening visit, and before PET and MRI scan. 7) for patients, a Diagnostic and Statistical Manual of Mental Disorders (DSM-IV) diagnosis of a schizophrenia according to the *Structured Clinical Interview of DSM-IV-TR Axis I Disorders-Patient Edition*.

Exclusion criteria for all participants: 1) ages <18 or >40; 2) a history of a head injury resulting in loss of consciousness; 3) personal history of serious medical illness; 4) contraindication to MRI scanning (e.g., metallic implants, claustrophobia, inability to lie comfortably for 90 minutes); 5) radiation exposure that would take total exposure (including participation in this study) to >10mSv in 12 months; 6) current or lifetime history of substance use or dependence as determined by the Structured Clinical Interview for DSM-IV-TR (SCID-I/P); 7) screened positive for any of the following substances (except cannabis) on a multi-panel urine drug screen detecting the following substance; amphetamine (300ng/ml cut off), cocaine (150 ng/ml cut off), ketamine (1000 ng/ml cut off), cannabis (50ng/ml cut off), methamphetamine (300 ng/ml cut off), opiates (2000 ng/ml cut off) (SureScreen Diagnostics, Derby); 8) current or recent use (no use within 3 months) of histaminergic drugs including, but not limited to, drugs with H3 affinity such as pitolisant, and other antihistaminergic drugs); 9) donation of blood or blood products in excess of 500ml within any 60 day period prior to the present study; 10) for healthy volunteers, a lifetime history of an Axis-I psychiatric disorder (DSM-IV) or confirmed diagnosis of Axis-I disorder in 1^st^ degree relatives.

**eMethods 2.** TMT administration

We applied the Trail Making Test (TMT) in this study. Poorer performance in TMT has been associated with altered functioning of the DLPFC in patients with psychotic illnesses (Hubl et al., 2018; Veselinović et al., 2018). Therefore, the DLPFC was chosen as a region of interest to correlate with TMT performance. Part A of the test requires the participant to search for and connect (by drawing a line) irregularly arranged targets containing numbers in ascending order. In part B, participants must connect consecutively numbered and lettered targets by alternating between two sequences (e.g 1-A-2-B-3 etc). The time taken to complete Part A and B of the TMT is recorded in seconds. TMT-A involves attention, visual search, motor functioning and working memory, whilst TMT-B places greater demands on executive functioning (Fujiki et al., 2013).

**eMethods 3:** PET Imaging Acquisition Parameters

All participants underwent a dynamic, continuous 90 minutes PET scan, following a bolus of [^11^C]MK-8278 diluted to 20ml with 0.9% NaCl normal saline was manually injected over ~20 seconds. All scans took place at the same time, in the late morning (10:00-13:00). Prior to PET acquisition, a low-dose CT topogram (0.36 mSv) was performed for attenuation correction during the PET image reconstruction. PET scans were acquired in list mode for 90 minutes using a Siemens BioGraph 6 HiRez PET-CT scanner (Siemens, Erlangen, Germany). PET emission data were corrected for attenuation and scatter and reconstructed using Fourier rebinning and 2D filtered back projection with a 2.0 mm kernel Ramp filter, into 26 dynamic frames (8x15s,3x60s,5x120s,5x300s,5x600s). The final reconstructed volume had voxel dimensions of 2.051 × 2.051 × 2.000 mm^3^.

In parallel to the PET acquisition, continuous arterial sampling using a blood sampler (Allogg ABSS (Allogg AB, Mariefred, Sweden, <http://www.allogg.se/> )) was performed for the first 15 minutes along with 12 discrete samples (5, 10, 15, 20, 25, 30, 40, 50, 60, 70, 80 and 90 minutes post injection). This was done to obtain individualised [^11^C]MK-8278 parent plasma input functions for each participant after correction for the radioactivity in blood cells and radioactive metabolites. To determine the plasma tracer radioactivity and to correct for the radiolabeled metabolites the plasma-over-blood (POB = concentration radiotracer in plasma/ concentration radiotracer in whole blood) and the parent plasma fraction (PPf = fraction of authentic radiotracer in plasma over the total plasma radioactivity) were defined for each PET acquisition.

**eMethods 4:** PET Image Analysis: Preprocessing Methods

### *Data pre-processing*

Data pre-processing was performed using a combination of Statistical Parametric Mapping 12 (<http://www.fil.ion.ucl.ac.uk/spm>) and FSL (<http://www.fsl.fmrib.ox.ac.uk/fsl>) functions, as implemented in MIAKAT (<http://www.imanova.co.uk)>. Motion correction was applied for all PET scans.

Attenuated corrected frames were realigned to a single “reference” frame, by employing a mutual information algorithm, creating a movement-corrected dynamic image, which was then used in the analysis. Realigned frames were then summated to create an individual motion-corrected reference map for the brain tissue segmentation. Specifically, individual T1-weighted MR images were co-registered to the PET image using rigid body transformation. Normalisation parameters were obtained by warping the co-registered structural MRI to MNI space (International Consortium for Brain Mapping ICBM/MNI) using bias-corrected segmentation in SPM12. The inverse of these parameters was used to fit the Clinical Imaging Centre (CIC) atlas to each individual PET scan (Tziortzi et al., 2011). For bilateral regions, left and right hemispheres were analysed in combination.

*Blood data processing*

Blood input functions (both whole blood data and plasma data), POB and PPf modelling were performed using MultiBlood, a unified framework for the arterial data modelling to achieve an accurate and fully automated description of the plasma tracer kinetics (Tonietto et al., 2015). The pipeline employed pursuit techniques for estimating both radiometabolites and parent concentration models from the raw plasma measurements, allowing the resulting algorithm to be both robust and flexible to the different quality of data available. Examples of blood data fit are reported in eFigure 1 while the distribution of parent-over-whole blood (POB) activity concentration ratio and parent plasma fraction (PPf) samples are reported in eFigure 2.

*Kinetic analysis*

For the region-of-interest analysis, we implemented the standard two-tissue compartmental model, expanding the previous analysis to describe [^11^C]MK-8278 brain kinetics (Van Laere et al., 2014). Identification of model parameters were done using nonlinear estimator (matlab lsqnonlin.m) and weighting each data point for the inverse of its error variance. The blood volume parameter (Vb) was fixed at 5% to reduce the error induced by the noisy blood input functions into the parameter estimates.

For ROI analysis, our primary endpoint was [^11^C]MK-8278 uptake for DLPFC and striatum. These areas were chosen given the prior findings (Jin et al., 2009) and evidence implicating the striatum in the pathophysiology of psychotic symptoms alongwith high density of H3R in this region that effect dopaminergic signalling (Alfaro-Rodriguez et al., 2013; McCutcheon et al., 2019; Ryu et al., 1994, 1996). The ROIs were obtained from the CIC atlas, a neuroanatomical atlas (Tziortzi et al., 2011). Using Statistical Parametric Mapping 12 (SPM12; version 6684)(The FIL Methods Group, 2014) gray matter masks were obtained by binarising segmented gray matter from T1-weighted images and applying this to the CIC atlas.

*Quality control*

The outputs of the image analysis were manually controlled for by experienced PET modelers. Specifically we tested that 1) brain extraction did not include loss of brain or excess of non-brain tissue, 2) GM, WM and CSF were properly extracted, 3) both MNI structural template and CIC atlas were aligned to individual MRI, 4) PET frames were realigned to the same space correcting for subject inter-frame motion, 5) the fitting of the blood input function was physiological (e.g. no negative values), and 6) the fitting of brain PET data provided by kinetic modelling was physiological (i.e. 2TCM). Those scans failing any of point 1 to 6 were labelled as QC failure and excluded from the analysis. All the scans (24/24) passed this quality control assessment.

**eMethods 5:** [^11^C]MK-8278 Uptake: Kinetic Modelling Validation

Two tissue compartmental (2TCM) model demonstrated good fit of the data, both in patients and controls (see eFigure 3). Compared to standard one tissue compartmental model (1TCM), 2TCM provided superior fitting performance and lower Akaike information index estimates for 77% of the cases analysed (all ROIs and all subjects).

V_T_ estimates from Logan graphical analysis demonstrated to be significantly correlated with 2TCM-based V_T_ estimates in all ROIs. The highest correlation value was measure for DLPFC (Pearson’s r = 0.92).

**eMethods 6:** Movement Parameters

Cumulative movement (CM) was defined the sum of frame-by-frame Euclidean distance, calculated by the frame realignment during PET motion correction. This parameter was higher in patients than controls (CM controls: 14±4mm, CM patients: 21±8mm, p=0.03) but no correlation was found between CM and V_T_ estimates in any of the ROIs considered. Thus, we excluded CM for further statistical analysis.

**eMethods 7:** [^11^C]MK-8278 blood uptake: cross-sectional comparison

There were no differences between groups in term of tracer activity in blood or plasma. Similarly, metabolite fractions were consistent across groups. There was 9% reduction in [^11^C]MK-8278 plasma protein binding (fp) in patients as compared to controls (fp controls: 41%±2%, fp patients: 37%±3%, p<0.01). However, no correlation was found between fp and V_T_ estimates in any of the ROIs consider. Thus, we excluded fp for further statistical analysis.

**eMethods 8:** Exploratory analyses

Detailed mapping of H3R with autoradiography, has identified high density of the receptor in the hippocampus (Chazot et al., 2001; Pillot et al., 2002). These receptors regulate synaptic transmission of hippocampal circuits which may affect cognitive processes including episodic memory (Brown and Haas, 1999; Brown and Reymann, 1996; Takei et al., 2017). This is supported by evidence from animal models of cognitive impairment associated with schizophrenia (CIAS) using scopolamine, where H3R antagonists acted to improve object recognition in rats (Giovannini et al., 1999). Therefore, we have included an exploratory hypothesis that hippocampal [^11^C]MK-8278 V_T_ would be inversely correlated with performance in an episodic memory task.

To assess episodic memory, we employed the Rey Auditory Verbal Learning Test (RAVLT) (Schmidt, 1996). This task assesses verbal episodic memory (Morrison et al., 2018; Schoenberg et al., 2006), and is a well-established tool for assessing cognitive function in schizophrenia (Zaytseva et al., 2018). The participant is instructed that they will hear a list of 15 nouns, which they will be asked to repeat once the assessor has completed reading them out. This is then repeated 4 times (A1-A5), after which an interference list of 15 is introduced (B1). The participant will then be asked to recall the original list without the assessor repeating the words (A6). Finally, the participant will be asked the recall the initial list of words after 20 minutes (A7). According to methodology of previous studies, we utilised the results from the A1, sum total A1-A5, B1, A6, and A7 as components of the task to specifically test for episodic memory (Lezak et al., 2004; Mensebach et al., 2009; Moritz et al., 2001).

To investigate the relationship between hippocampal [^11^C]MK-8278 V_T_ and episodic memory, Pearson’s correlation coefficients were employed, as both sets of data were normally distributed.

**eTable 1:** PET data – Volume of distribution (V_T_) in ROIs.

|  | **Patient V_T_, ml/cm^3^, mean (SD)** | **Control V_T_, ml/cm^3^, mean (SD)** |
| --- | --- | --- |
| **DLPFC** | 10.9 (2.2) | 11.2 (2.7) |
| **Striatum** | 23.6 (4.7) | 24.6 (4.4) |
| **Hippocampus** | 10.6 (1.3) | 11.2 (1.6) |
| **Nucleus Accumbens** | 26.0 (4.5) | 28.1 (3.6) |
| **Thalamus** | 11.0 (1.4) | 11.6 (1.6) |
| **Frontal Lobe** | 11.2 (2.8) | 12.0 (2.8) |
| **Anterior Cingulate** | 14.6 (2.2) | 15.7 (2.5) |
| **Parietal Lobe** | 9.8 (1.8) | 10.6 (1.9) |
| **Insular Cortex** | 13.8 (2.2) | 15.0 (2.5) |
| **Temporal Lobe** | 10.5 (1.8) | 11.3 (1.9) |
| **Occipital Lobe** | 8.9 (1.2) | 9.5 (1.4) |
| **Pons** | 6.2 (0.8) | 6.7 (0.9) |

**eTable 2**. Experimental Parameters

|  | Healthy volunteers/  Mean (SD) | Patients with FEP/  Mean (SD) | Mann-Whitney U | df | p |
| --- | --- | --- | --- | --- | --- |
| N | 12 | 12 |  |  |  |
| Age (years) | 31 (11) | 31 (12) | 67.50 |  | 0.80 |
| Weight (Kg) | 73.1 (11.8) | 79.7 (12.8) | t = -1.26 | 22 | 0.22 |
| Dose (MBq) | 275.63 (10.29) | 251.55 (17.36) | 11.00 |  | <0.001 |
| Injected mass (μg) | 3.84 (1.85) | 3.74 (1.62) | 74.00 |  | 0.93 |
| Specific activity (GBq/μmol) | 34.56 (12.85) | 31.00 (9.99) | t = 0.72 | 22 | 0.28 |
| fp | 0.41 (0.02) | 0.37 (0.02) | t = 4.49 | 22 | <0.001 |
| Total motion during 90min scan (mm) | 13.86 (4.18) | 20.61 (8.32) | 108.00 |  | 0.04 |
| Max interframe motion (mm) | 2.47 (1.12) | 3.14 (1.44) | t = -1.22 | 22 | 0.24 |
| AUC Cp | 22.95 (3.71) | 23.48 (2.94) | 83.00 |  | 0.55 |
| AUC Cp (peak) | 9.65 (1.71) | 9.72 (1.67) | 55.00 |  | 0.35 |
| PPf - 5min | 0.85 (0.03) | 0.86 (0.03) | 61.00 |  | 0.55 |
| PPf - 90 min | 0.22 (0.07) | 0.23 (0.04) | 70.00 |  | 0.93 |

**eTable 3:** RAVLT performance

| RAVLT task component | Healthy volunteers/  Mean (SD) | Patients with FEP/  Mean (SD) | *t* | df | p |
| --- | --- | --- | --- | --- | --- |
| A1 | 6.83 (1.47) | 4.50 (1.98) | 3.28 | 22 | 0.003 |
| Sum A1-A5 | 50.50 (8.27) | 40.67 (10.24) | 2.59 | 22 | 0.02 |
| B1 (interference trial) | 5.33 (1.61) | 4.42 (2.46) | 1.08 | 22 | 0.29 |
| A6 (free recall) | 10.67 (3.17) | 7.75 (3.05) | 2.30 | 22 | 0.03 |
| A7 (delayed recall) | 10.67 (3.50) | 7.67 (3.65) | 2.06 | 22 | 0.03 |

**eTable 4:** Episodic memory and hippocampal [^11^C]MK-8278 V_T_

| Control group Pearson correlation / Pearson’s r (p) | |  | Patient group Pearson correlation / Pearson’s r (p) | |
| --- | --- | --- | --- | --- |
|  | Hippocampal V_T_ |  |  | Hippocampal V_T_ |
| A1 | r = -0.14 (p = 0.66) |  | A1 | r = -0.09 (p = 0.79) |
| Sum A1-A5 | r = -0.26 (p = 0.41) |  | Sum A1-A5 | r = -0.001 (p = 0.999) |
| B1 (interference trial) | r = -0.03 (p = 0.91) |  | B1 (interference trial) | r = 0.22 (p = 0.49) |
| A6 (free recall) | r = -0.22 (p = 0.49) |  | A6 (free recall) | r = -0.08 (p = 0.80) |
| A7 (delayed recall) | r = -0.30 (p = 0.34) |  | A7 (delayed recall) | r = -0.07 (p = 0.83) |

**eFigure 1:** Whole blood and parent plasma data of radiotracer activity over time.

1. B)

**
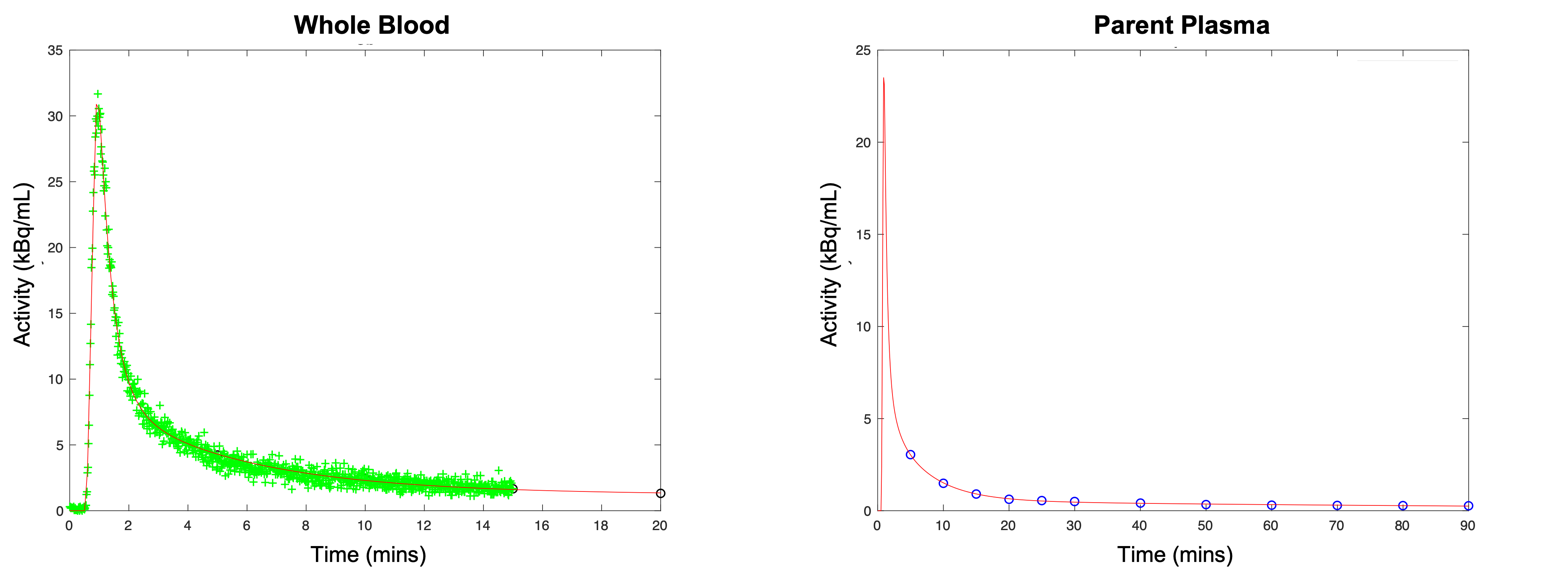
**

(A) The continuous and discrete datasets, from arterial blood sampling, were used to form a whole blood activity curve, covering the duration of the scan. The plots indicate each sample across both groups which were averaged to develop a mean activity curve. (B) The whole blood activity curve was used to calculate the concentration of the non-metabolised tracer in the arterial plasma, which is defined in the plasma time activity curve above.

**eFigure 2:** Plasma-over-Blood (POB) activity concentration ratio and Parent Plasma Fraction (PPf) activity over time

1. B)

**
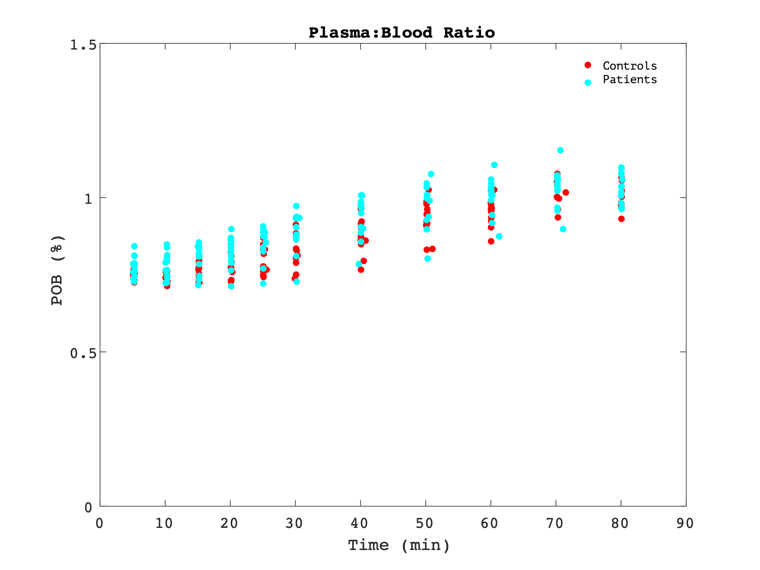

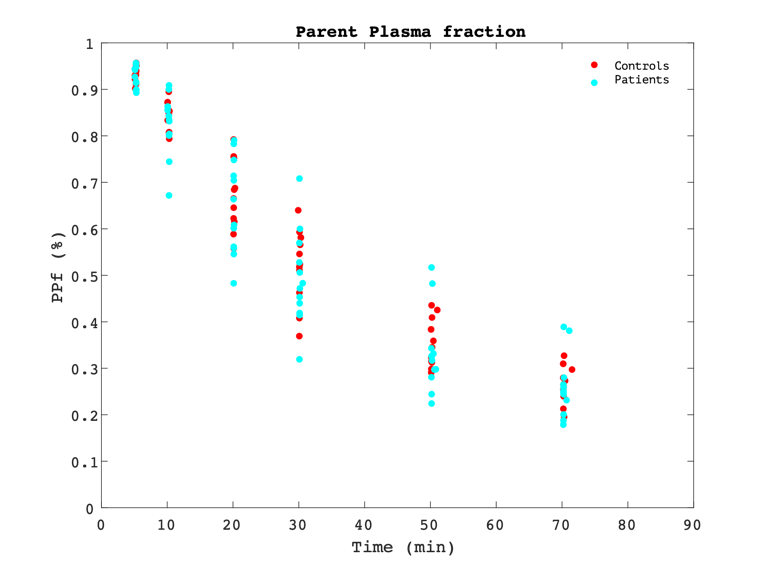
**

(A) Radioactivity concentrations in discrete plasma samples were divided by the corresponding whole-blood samples to form POB data, which are displayed above. (B) Subsequently, PPf data was calculated for both patients and controls.

**eFigure 3:** Time Activity Curves for a representative control and patient, including 1-Tissue Compartment Model (1TCM) and 2-Tissues Compartment Model (2TCM).


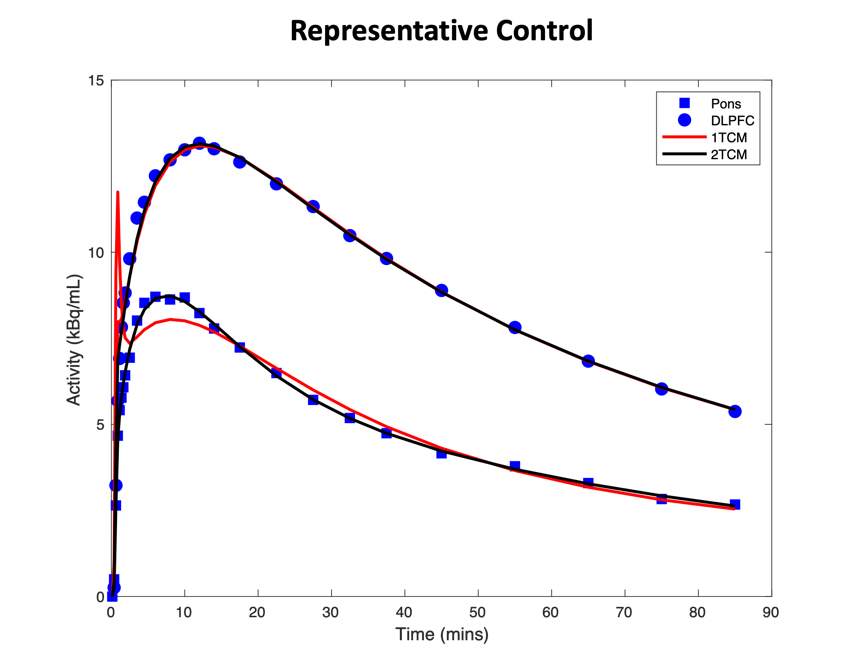

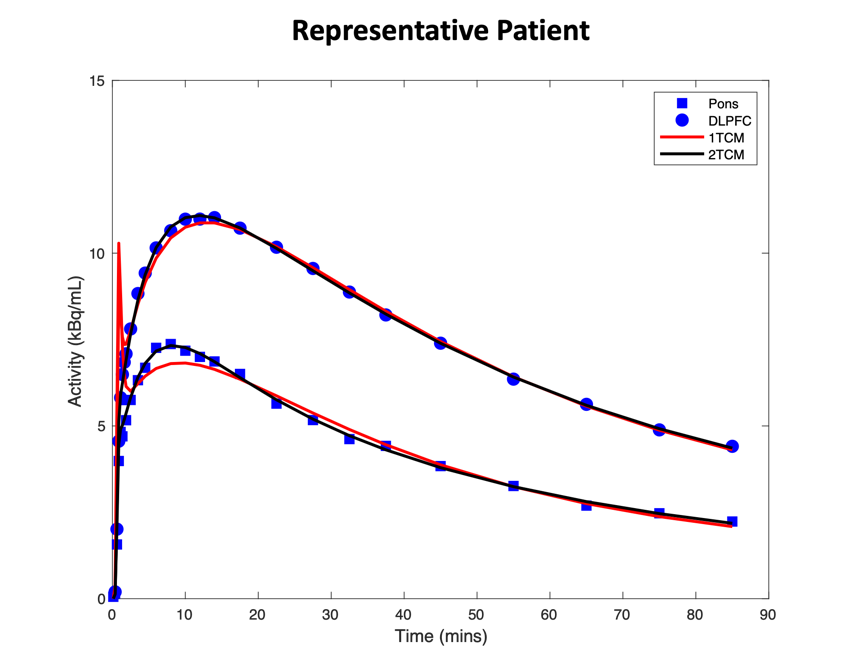


Regions indicated in these graphs include the DLPFC and pons, which were the primary ROI and region of lowest volume of distribution, respectively. Our modelling indicated that 2TCM had a greater fit for the current data, compared to 1TCM.

**eFigure 4:** Relationship between ROIs [^11^C]MK-8278 V_T_ and PANSS positive symptom severity

1. **B)**

**
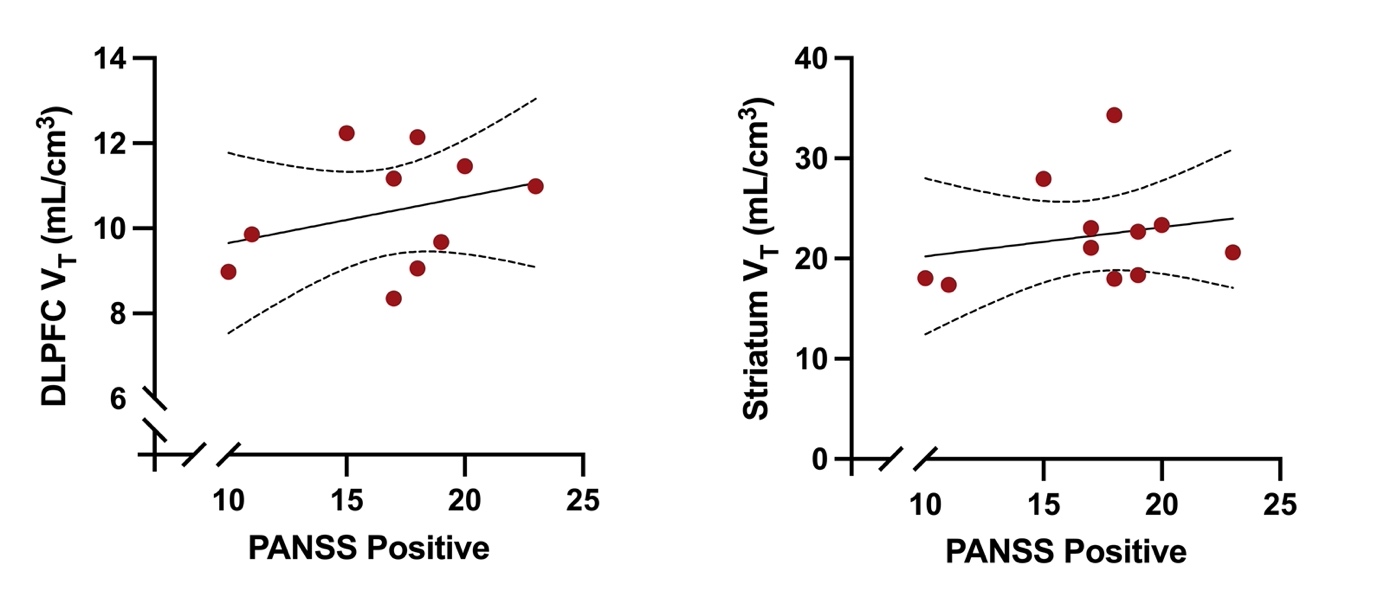
**

**A)** DLPFC [^11^C]MK-8278 V_T_ and PANSS positive symptom severity (Pearson r = 0.31, p = 0.39). **B)** Striatum [^11^C]MK-8278 V_T_ and PANSS positive symptom severity (Pearson r = 0.22, p = 0.52). Dashed line represents 95% confidence interval.

**eFigure 5:** Relationship between ROIs [^11^C]MK-8278 V_T_ and PANSS negative symptom severity

1. **B)**

**
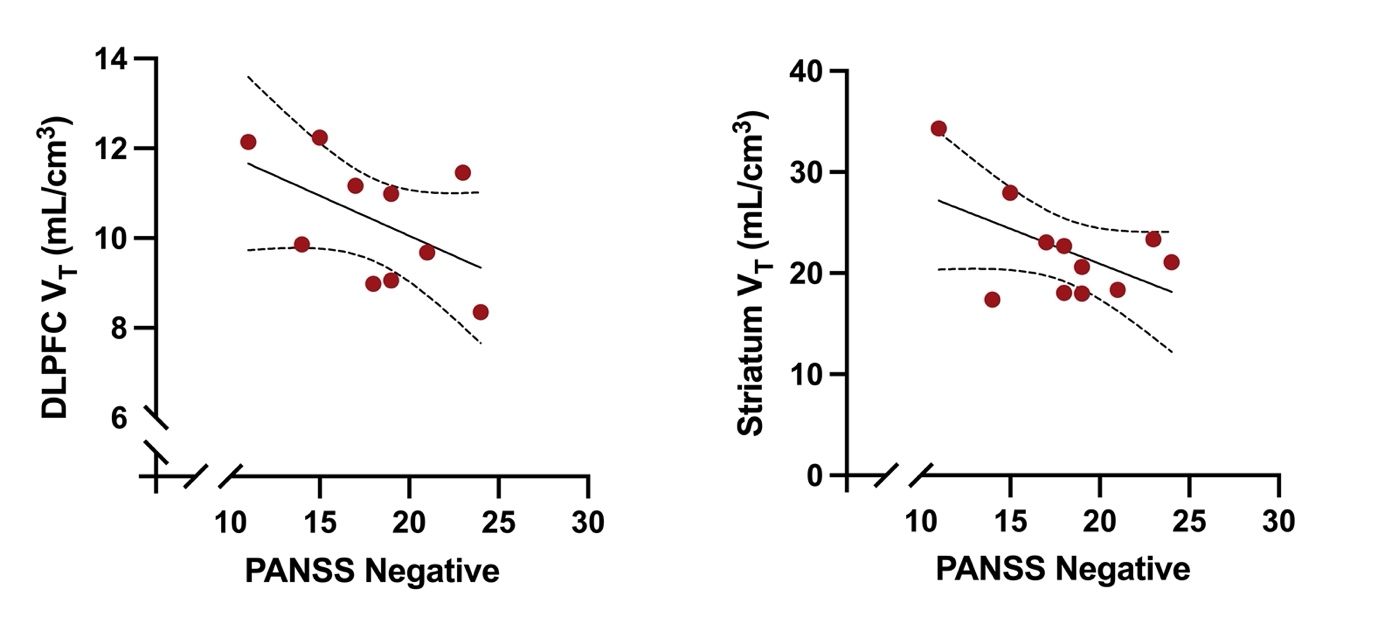
**

**A)** DLPFC [^11^C]MK-8278 V_T_ and PANSS negative symptom severity (Pearson r = -0.52, p = 0.12). **B)** Striatum [^11^C]MK-8278 V_T_ and PANSS negative symptom severity (Pearson r = -0.52, p = 0.10). Dashed line represents 95% confidence interval.

**eFigure 6:** Relationship between ROIs [^11^C]MK-8278 V_T_ and PANSS general symptom severity

1. **B)**


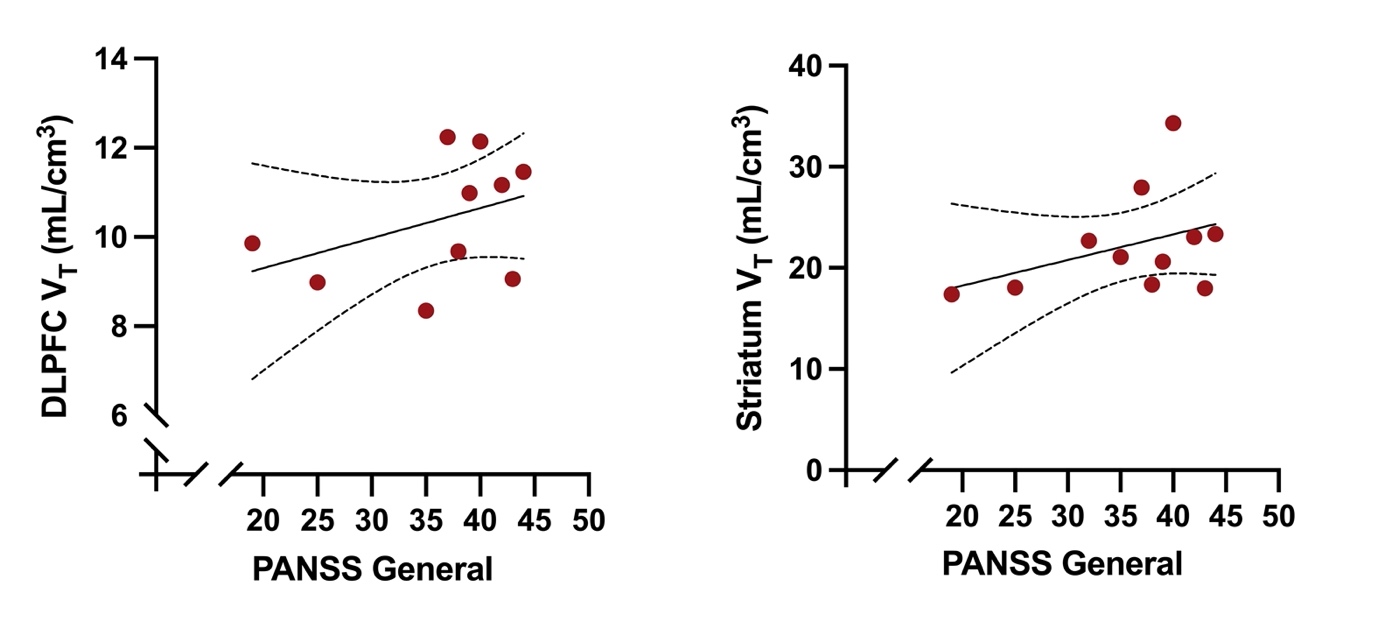


**A)** DLPFC [^11^C]MK-8278 V_T_ and PANSS negative symptom severity (Pearson r = 0.39, p = 0.26). **B)** Striatum [^11^C]MK-8278 V_T_ and PANSS negative symptom severity (Pearson r = 0.39, p = 0.24). Dashed line represents 95% confidence interval.

**eReferences:**

Alfaro-Rodriguez A, Alonso-Spilsbury M, Arch-Tirado E, et al. (2013) Histamine H3 receptor activation prevents dopamine D1 receptor-mediated inhibition of dopamine release in the rat striatum: a microdialysis study. *Neuroscience letters* 552: 5–9. DOI: 10.1016/j.neulet.2013.07.026.

Brown RE and Haas HL (1999) On the mechanism of histaminergic inhibition of glutamate release in the rat dentate gyrus. *The Journal of physiology* 515 ( Pt 3)(Pt 3). England: 777–786. DOI: 10.1111/j.1469-7793.1999.777ab.x.

Brown RE and Reymann KG (1996) Histamine H3 receptor-mediated depression of synaptic transmission in the dentate gyrus of the rat in vitro. *The Journal of physiology* 496 ( Pt 1(Pt 1): 175–184. DOI: 10.1113/jphysiol.1996.sp021675.

Chazot PL, Hann V, Wilson C, et al. (2001) Immunological identification of the mammalian H3 histamine receptor in the mouse brain. *Neuroreport* 12(2). England: 259–262. DOI: 10.1097/00001756-200102120-00016.

Giovannini MG, Bartolini L, Bacciottini L, et al. (1999) Effects of histamine H3 receptor agonists and antagonists on cognitive performance and scopolamine-induced amnesia. *Behavioural brain research* 104(1–2). Netherlands: 147–155. DOI: 10.1016/s0166-4328(99)00063-7.

Jin C, Anichtchik O and Panula P (2009) Altered histamine H3 receptor radioligand binding in post-mortem brain samples from subjects with psychiatric diseases. *British Journal of Pharmacology* 157(1): 118–129. DOI: 10.1111/j.1476-5381.2009.00149.x.

Lezak MD, Howieson DB, Loring DW, et al. (2004) *Neuropsychological Assessment*. Oxford University Press, USA.

McCutcheon RA, Abi-Dargham A and Howes OD (2019) Schizophrenia, Dopamine and the Striatum: From Biology to Symptoms. *Trends in Neurosciences* 42(3). Elsevier: 205–220. DOI: 10.1016/j.tins.2018.12.004.

Mensebach C, Beblo T, Driessen M, et al. (2009) Neural correlates of episodic and semantic memory retrieval in borderline personality disorder: An fMRI study. *Psychiatry Research: Neuroimaging* 171(2): 94–105. DOI: https://doi.org/10.1016/j.pscychresns.2008.02.006.

Moritz S, Heeren D, Andresen B, et al. (2001) An analysis of the specificity and the syndromal correlates of verbal memory impairments in schizophrenia. *Psychiatry Research* 101(1): 23–31. DOI: https://doi.org/10.1016/S0165-1781(00)00241-9.

Morrison RL, Pei H, Novak G, et al. (2018) A computerized, self-administered test of verbal episodic memory in elderly patients with mild cognitive impairment and healthy participants: A randomized, crossover, validation study. *Alzheimer’s & Dementia: Diagnosis, Assessment & Disease Monitoring* 10: 647–656. DOI: https://doi.org/10.1016/j.dadm.2018.08.010.

Pillot C, Heron A, Cochois V, et al. (2002) A detailed mapping of the histamine H3 receptor and its gene transcripts in rat brain. *Neuroscience* 114(1): 173–193. DOI: https://doi.org/10.1016/S0306-4522(02)00135-5.

Ryu JH, Yanai K, Iwata R, et al. (1994) Heterogeneous distributions of histamine H3, dopamine D1 and D2 receptors in rat brain. *NeuroReport* 5(5). Available at: https://journals.lww.com/neuroreport/Fulltext/1994/01000/Heterogeneous_distributions_of_histamine_H3,.22.aspx.

Ryu JH, Yanai K, Zhao X-L, et al. (1996) The effect of dopamine D1 receptor stimulation on the up-regulation of histamine H3-receptors following destruction of the ascending dopaminergic neurones. *British Journal of Pharmacology* 118(3). John Wiley & Sons, Ltd (10.1111): 585–592. DOI: 10.1111/j.1476-5381.1996.tb15441.x.

Schmidt M (1996) *Rey Auditory Verbal Learning Test: A Handbook*. Western Psychological Services Los Angeles, CA.

Schoenberg MR, Dawson KA, Duff K, et al. (2006) Test performance and classification statistics for the Rey Auditory Verbal Learning Test in selected clinical samples. *Archives of Clinical Neuropsychology* 21(7): 693–703. DOI: https://doi.org/10.1016/j.acn.2006.06.010.

Takei H, Yamamoto K, Bae Y-C, et al. (2017) Histamine H3 Heteroreceptors Suppress Glutamatergic and GABAergic Synaptic Transmission in the Rat Insular Cortex . *Frontiers in Neural Circuits* . Available at: https://www.frontiersin.org/article/10.3389/fncir.2017.00085.

The FIL Methods Group (2014) *Statistical Parametric Mapping Software*. Institute of Neurology, University College London.

Tonietto M, Veronese M, Rizzo G, et al. (2015) Improved models for plasma radiometabolite correction and their impact on kinetic quantification in PET studies. *Journal of cerebral blood flow and metabolism : official journal of the International Society of Cerebral Blood Flow and Metabolism* 35(9). SAGE Publications: 1462–9. DOI: 10.1038/jcbfm.2015.61.

Tziortzi AC, Searle GE, Tzimopoulou S, et al. (2011) Imaging dopamine receptors in humans with [11C]-(+)-PHNO: dissection of D3 signal and anatomy. *NeuroImage* 54(1). United States: 264–277. DOI: 10.1016/j.neuroimage.2010.06.044.

Van Laere KJ, Sanabria-Bohorquez SM, Mozley DP, et al. (2014) 11C-MK-8278 PET as a Tool for Pharmacodynamic Brain Occupancy of Histamine 3 Receptor Inverse Agonists. *Journal of Nuclear Medicine* 55(1): 65–72. DOI: 10.2967/jnumed.113.122515.

Zaytseva Y, Fajnerová I, Dvořáček B, et al. (2018) Theoretical Modeling of Cognitive Dysfunction in Schizophrenia by Means of Errors and Corresponding Brain Networks. *Frontiers in psychology* 9. Switzerland: 1027. DOI: 10.3389/fpsyg.2018.01027.
